# Waterpipe cafes in Baltimore, Maryland: Carbon monoxide, particulate matter, and nicotine exposure

**DOI:** 10.1038/jes.2014.19

**Published:** 2014-04-16

**Authors:** Christine M Torrey, Katherine A Moon, D' Ann L Williams, Tim Green, Joanna E Cohen, Ana Navas-Acien, Patrick N Breysse

**Affiliations:** 1 Department of Environmental Health Sciences, Johns Hopkins University Bloomberg School of Public Health, Baltimore, Maryland, USA; 2 Institute for Global Tobacco Control, Department of Health, Behavior and Society, Johns Hopkins Bloomberg School of Public Health, Baltimore, Maryland, USA; 3 Department of Epidemiology and Welch Center for Prevention, Epidemiology and Clinical Research, Johns Hopkins Bloomberg School of Public Health, Baltimore, Maryland, USA

**Keywords:** waterpipe, hookah, second hand smoke, particulate matter, carbon monoxide, nicotine

## Abstract

Waterpipe smoking has been growing in popularity in the United States and worldwide. Most tobacco control regulations remain limited to cigarettes. Few studies have investigated waterpipe tobacco smoke exposures in a real world setting. We measured carbon monoxide (CO), particulate matter (PM)_2.5_, and airborne nicotine concentrations in seven waterpipe cafes in the greater Baltimore area. Area air samples were collected between two and five hours, with an average sampling duration of three hours. Waterpipe smoking behaviors were observed at each venue. Indoor air samplers for CO, PM_2.5_, and airborne nicotine were placed in the main seating area 1–2 m above the floor. Indoor airborne concentrations of PM_2.5_ and CO were markedly elevated in waterpipe cafes and exceeded concentrations that were observed in cigarette smoking bars. Air nicotine concentrations, although not as high as in venues that allow cigarette smoking, were markedly higher than in smoke-free bars and restaurants. Concentrations of PM approached occupational exposure limits and CO exceeded occupational exposure guidelines suggesting that worker protection measures need to be considered. This study adds to the literature indicating that both employees and patrons of waterpipe venues are at increased risk from complex exposures to secondhand waterpipe smoke.

## INTRODUCTION

Tobacco-related research and control efforts have generally focused on cigarettes, while other forms of tobacco products and tobacco uses are common in many countries. Waterpipe cafes and waterpipe tobacco smoking have been growing in popularity in the United States and worldwide, particularly with young adults.^1, 2, 3, 4^ Despite this increase, most tobacco control regulations remain limited to cigarettes. Furthermore, some cities and states specifically exempt waterpipe smoking.^3^ These exemptions may in part be due to the perception that waterpipe smoking is less harmful than cigarette smoking because of the pervasive belief that water removes dangerous tobacco components or because the smell produced seems less noxious.^3, 4^

Waterpipe (variously referred to as hookah, hukkah, narghile, chicha, or shisha) generates mainstream and side-stream smoke differently than cigarettes because waterpipe combustion occurs at lower temperatures than cigarette combustion. In a waterpipe smoking session, moistened tobacco is placed in the head of the waterpipe, perforated foil is placed over the head, and charcoal is loaded onto the foil and lit. The smoker inhales through the hose drawing smoke from the charcoal and tobacco combustion through the water. As a result of this design, there is incomplete combustion of the tobacco. Both the incomplete combustion of the tobacco and the charcoal briquette contribute to the levels of carbon monoxide (CO) and particulate matter (PM) in the air.^5, 6^

Laboratory and controlled studies have shown that mainstream and side-stream waterpipe smoke contain large quantities of nicotine, fine and ultrafine PM, CO, polycyclic aromatic hydrocarbons, volatile aldehydes, and phenolic compounds.^6, 7, 8, 9, 10, 11^ Smoke from a single waterpipe session has been shown to emit approximately four times the carcinogenic PAH, four times the volatile aldehydes, and 30 times the CO of a single cigarette.^8^ Biomonitoring studies of waterpipe usage in controlled settings have documented elevated exhaled CO and blood nicotine levels, as well as elevated concentrations of blood carboxyhemoglobin and urinary excretion of 4-(methylnitrosamino)-1-(3-pyridyl)-1- butanol and polycyclic aromatic hydrocarbons.^5, 12, 13^

Few studies have investigated waterpipe tobacco smoke exposures in a real world setting.^14, 15, 16^ For example, a recent study of indoor air quality in waterpipe cafes and cigarette-permitting restaurants in Virginia found that fine PM concentrations observed in waterpipe cafes were 3.2 times greater than restaurants, which permitted cigarette smoking.^15^ Little is known about waterpipe-related toxicant concentrations in real world settings. To provide information on air quality in waterpipe cafes and to assess exposure to waterpipe tobacco toxicants in a real world setting, we measured CO, PM <2.5 *μ*m in diameter (PM_2.5_), and airborne nicotine concentrations in waterpipe cafes in the greater Baltimore area.

## MATERIALS AND METHODS

### Study Population and Data Collection

Seven waterpipe cafes in the greater Baltimore area were surveyed between December 2011 and August 2012. Two venues were surveyed twice for a total of nine study visits. These venues represent all open establishments in the greater Baltimore area at the time of the survey. Venues were visited during peak occupancy times on Friday or Saturday night between 2000 and 0200 hours. Each visit included indoor air sampling, and recording of fixed venue characteristics and waterpipe smoking behaviors. Air samples were collected for periods of time ranging between two and five hours, with an average sampling duration of three hours. Fixed venue characteristics included venue size (volume) and ventilation sources. Ventilation sources, such as open doors or windows and presence of a kitchen with a functioning stove or oven, were documented to ascertain whether air concentrations of PM_2.5_ and CO could be from combustion sources besides waterpipe smoking. Waterpipe smoking behaviors were observed upon entering the venue and at ∼15-minute intervals thereafter until exiting the venue. We recorded the number of people, the number of lit waterpipes, the number of people actively smoking waterpipe, and the average number of people sharing a waterpipe.

### Indoor Air Sampling

Indoor air sampling included CO, PM_2.5_, and airborne nicotine. Air samplers were placed in the main seating area 1–2 m above the floor in backpacks or other bags in an effort not to disrupt the normal behaviors of patrons and employees. Sample locations were selected to provide representative estimates of indoor air concentrations within each venue. We collected background PM_2.5_ and CO samples outside the entrance of each venue for ∼10 min before and after each visit.

#### Carbon monoxide

We measured CO at 1-m intervals using a Lascar USB direct reading and data logging EL-USB-CO300 instrument (0 p.p.m. to 300 p.p.m. Carbon Monoxide USB data logger; Lascar Electronics, Erie, PA, USA). The EL-USB-CO300 monitor is calibrated annually by the manufacturer. Calibration quality control was performed before fieldwork using a 146C Dynamic Gas Calibrator (Thermo Environmental Instruments, Franklin, MA, USA), fitted with a CO regulator, CO tank (Matheson TRI*GAS, Twinsburg, OH, USA), and a Zero air pump (GAST Manufacturing corporation, Benton Harbor, MI, USA). CO monitors were suspended in a large chamber and were exposed to five concentrations of CO (5 p.p.m., 10 p.p.m., 30 p.p.m., 40 p.p.m., and 50 p.p.m.) using the Dynamic Gas Calibrator. These measurements were found to be within 5% of the tested value. At six venues, we collected CO samples at two different locations simultaneously to assess spatial variability. These samples were in close agreement (10%) and were averaged to give a venue composite concentration. For CO concentrations below the level of detection, we imputed the concentrations as half of the level of detection (0.25 p.p.m.).

#### Particulate matter

We measured PM_2.5_ at 1-m intervals using a TSI SidePak AM510 Personal Aerosol Monitor (SidePak; TSI, Shoreview, MN, USA). In one venue, we collected a duplicate PM_2.5_ sample. Data from each visit were downloaded using the TSI Trackpro V3.4.1 software (TSI, Shoreview, MN, USA). In a subset of five venues, we also collected gravimetric PM_2.5_ using a Personal Environmental Monitor (SKC model 761-203A; SKC, Eighty Four, PA, USA) with Teflon filter (Pall Corporation, Port Washington, NY, USA) in conjunction with a BGI 400 Personal Sampling Pump with battery pack (BGI Incorporated, Waltham, MA, USA), set to sample at 4 l/min (26). PM_2.5_ filters were weighed before and after sampling in a temperature and humidity controlled environment on a Mettler Toledo XP2U balance (Mettler Toledo, Columbus, OH, USA). During data collection, we measured temperature and relative humidity every minute using a HOBO U10 Temperature Relative Humidity Data Logger (U10-003; Onset Computer Corporation, Bourne, MA, USA). Relative humidity never exceeded 60% and the median (25th, 75th percentile) temperature across all venues was 75 °F (70 °F, 82 °F).

#### Nicotine

We measured vapor-phase nicotine using a passive, diffusion-based sampler treated with sodium bisulfate.^17, 18^ Vapor-phase nicotine is commonly used as a marker of tobacco smoke exposure.^18^ For quality control purposes, we collected 10% field blanks and duplicate samples. The filters were extracted with an internal standard (Nicotinine-d3; Supelco, Bellefonte, PA, USA) and were analyzed using gas chromatography and mass spectrometry (GC-17/MS-QP5000; Shimadzu, Kyoto, Japan) in SIM and splitless mode. The gas chromatograph oven temperature was maintained at 50 °C for one minute, increased to 200 °C at a rate of 30 °C per minute, then increased by 15 °C per minute until 280 °C and held for one minute. Nicotine was separated using a capillary column (30 m × 0.25 mm internal diameter, 0.25-μm film thickness (Rtx–624, Restek, Bellefonte, PA, USA). Airborne concentrations of nicotine were calculated by dividing the amount of nicotine collected by each filter (*μ*g) by the volume of air sampled (m^3^). The volume of air sampled is equal to the total of sampling time multiplied by the effective flow rate (25 ml/min). Nicotine values below the analytically determined limit of quantitation were replaced with ½ the sample-specific limit of quantitation.

### Data Analysis

For each venue, PM_2.5_ and CO data logged before the recorded entry and exit times were coded as outdoor air and removed from the analyses of indoor air concentrations (a comparison of outdoor *vs* indoor air concentrations can be found in Supplementary Table 1).

SidePak PM_2.5_ measurements must be calibrated to the specific aerosol being sampled because the light-scattering properties of PM_2.5_ vary substantially with particle size and composition (28). We estimated a waterpipe-specific calibration factor for PM_2.5_ SidePak measurements by regressing gravimetric PM_2.5_ on arithmetic mean real-time PM_2.5_ concentrations. The estimated regression coefficient, 0.60, was then used as a calibration factor and applied to the SidePak PM_2.5_ data. Sensitivity analyses conducted by sequentially omitting each venue yielded relatively consistent results (in the range of 0.49–0.65). High humidity can influence reported PM concentrations from light-scattering devices. Because none of the venues had a relative humidity above 60%, we made no humidity adjustment.

For PM_2.5_ and CO, we evaluated the distributions using summary statistics and box plots. We assessed the relationship between PM_2.5_ and CO at each venue and between PM_2.5_ and nicotine across venues using Spearman correlation coefficients. The relationship between PM_2.5_ and CO was further evaluated using linear regression models, with log-transformed concentrations of both PM_2.5_ and CO due to right-skewed distributions.

Active smoker density, the number of burning cigarettes per 100 m^3^, is a common metric of secondhand smoke exposure. We calculated an active waterpipe density by determining the number of people actively smoking waterpipe per 100 m^3^.

All statistical analyses were performed with R Version 2.5.1 (R foundation for Statistical Computing, www.r-project.org, Vienna, Austria). *P*-values less than 0.05 were considered statistically significant.

## RESULTS

### Venue Characteristics and Waterpipe Smoking Observations

Venue characteristics and smoking observations are summarized in Table 1. Venues ranged in size from 32 m^2^ to 140 m^2^ (112 m^3^ to 490 m^3^) and most were poorly ventilated, with closed windows and doors that only opened intermittently. Only one venue (Venue #4) had a functioning kitchen, a potential alternative source of combustion byproducts.

Over the course of observation, venues varied greatly by number of people, number of waterpipes lit, and the number of people actively smoking waterpipes (Table 1). The mean SD number of people ranged from 10 (2) to 41 (2). The mean (SD) number of lit waterpipes ranged from 10 (7) to 14 (9), while the number of people actively smoking waterpipe ranged from 8 (5) to 14 (10). The mean (SD) number of people sharing waterpipes ranged from 3 (1) to 8 (1). The mean (SD) active waterpipe density (number of people actively smoking waterpipe per 100 m^3^) ranged from 2 (1) people in Venues #5 and #6 to 12 (9) people in the first visit to Venue #1, Venue #1-A (Table 1).

### Carbon Monoxide

**Figure 1:**
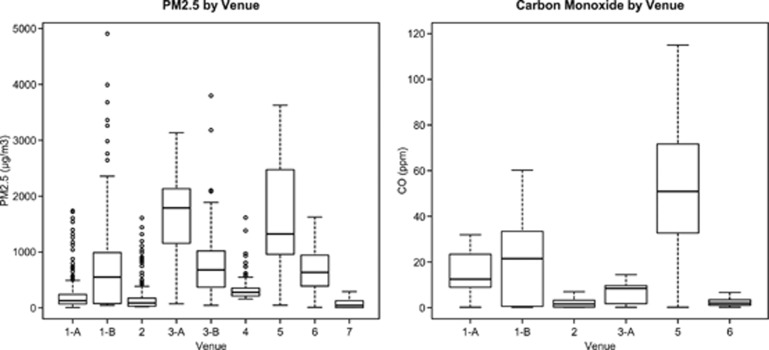
Box plots of particulate matter (PM)_2.5_ and carbon monoxide (CO) by Venue.

CO was only measured in six of the nine visits due to equipment malfunctioning. CO concentrations varied widely across venues and study visits (Table 2; Figure 1). The overall median (interquartile range) and mean (SD) CO concentrations were 9 (2,25) p.p.m. and 18 (13) p.p.m., respectively (Table 2). The highest mean CO concentration was 53 p.p.m. (Venue #5) and the lowest was 2 p.p.m. (Venue #2 and Venue #6). Maximum CO concentrations averaged 39 p.p.m. with a range of 7 p.p.m. to 115 p.p.m. (recorded at Venue #5).

Indoor mean CO concentrations were between 1.5- and 8.8-fold higher than the corresponding outdoor mean CO concentrations collected for approximately 10–15 min before entering each venue (Supplementary Table 1).

### Particulate Matter

**Figure 2:**
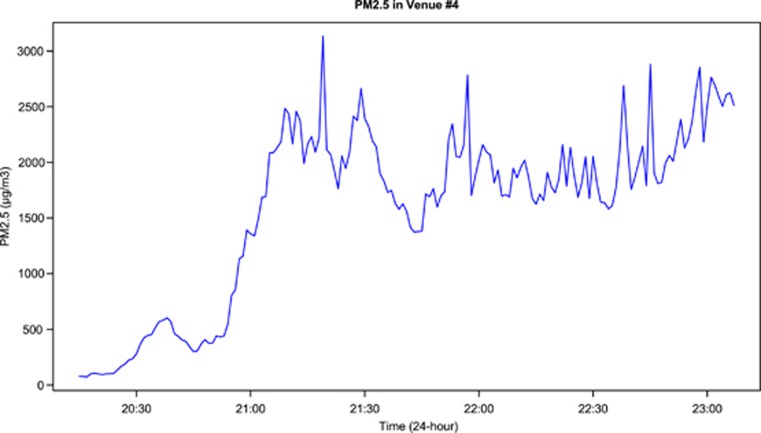
Real-time indoor air particulate matter (PM)_2.5_ concentrations in Venue #4 during an ∼3-h period.

Indoor PM_2.5_ concentrations also varied widely across venues and study visits (Table 2; Figure 1). The median (interquartile range) and mean (SD) concentrations of gravimetric-corrected real-time PM_2.5_ across all venues and visits were 374 (127–1032) *μ*g/m^3^ and 712 (785) *μ*g/m^3^, respectively (Table 2). An example plot of real-time PM_2.5_ *vs* time is shown in Figure 2. The lowest mean real-time PM_2.5_ concentration was 72 *μ*g/m^3^ in Venue #7. Maximum PM_2.5_ concentrations averaged 2483 *μ*g/m^3^ across all visits. The highest peak value of 4907 *μ*g/m^3^ was observed in Venue #1-B.

Indoor mean PM_2.5_ concentrations were between 1.3- and 24.1-fold higher than the corresponding outdoor mean PM_2.5_ concentrations collected for approximately 10–15 min before entering each venue (Supplementary Table 1).

### Airborne Nicotine

Airborne nicotine concentrations ranged from 0.77 *μ*g/m^3^ in Venue #2 to 1.9 *μ*g/m^3^ in Venue #3-A (first visit) (Table 2). The mean (SD) concentration of airborne nicotine across all venues was 1.42 (0.42) *μ*g/m^3^.

### Relationships Between Secondhand Smoke Constituents

**Figure 3:**
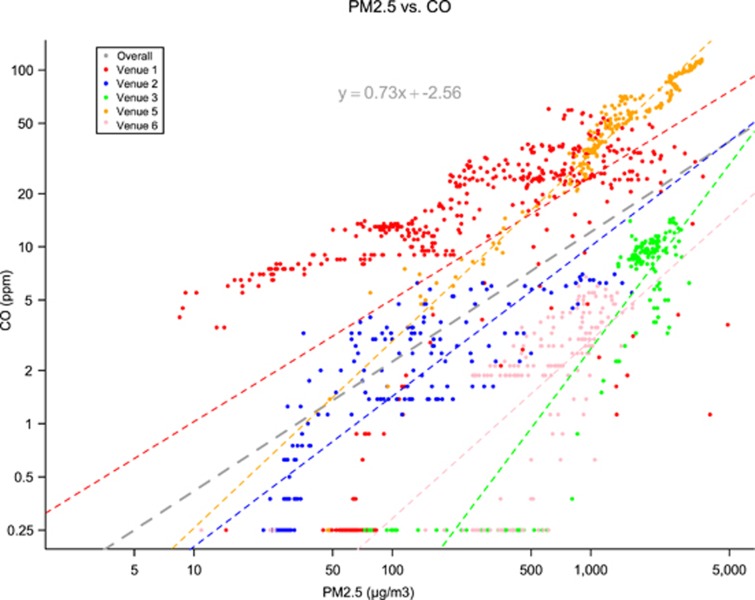
Scatterplot of particulate matter (PM)_2.5_ and carbon monoxide (CO). Multiple visits to a single venue were combined (Venue #1 and Venue #3). PM_2.5_ and CO measurements were log-transformed.

Real-time PM_2.5_ and CO concentrations were strongly correlated within each venue (Spearman correlation coefficients ranged between 0.72 and 0.97; all *P*-values <0.001) (Table 3). Overall, each 10 *μ*g/m^3^ increase in PM_2.5_ was associated with a 0.2-p.p.m. increase in CO (*P*<0.001) (Figure 3). This relationship was consistent across venues (Figure 3).

PM_2.5_ and nicotine concentrations were moderately correlated across venues. Correlations between nicotine and the arithmetic mean, geometric mean, median, and maximum real-time PM_2.5_ were 0.57 (*P*=0.12), 0.58 (*P*=0.11), 0.58 (*P*=0.11), and 0.48 (*P*=0.19), respectively.

## DISCUSSION

Indoor airborne concentrations of PM_2.5_ and CO were markedly elevated in waterpipe cafes from the Baltimore area, confirming that waterpipe smoking severely affects indoor air quality. Air nicotine concentrations, although not as high as in hospitality venues that allow cigarette smoking,^19^ were also elevated and markedly higher than in smoke-free bars and restaurants. This study is one of the few assessments of indoor air quality in water pipe cafes in the United States, and one of the first to include PM_2.5_, CO, and airborne nicotine.^16^

The mean PM_2.5_ concentrations measured in Baltimore water pipe venues, 712 *μ*g/m^3^, greatly and consistently exceeded the 24-h ambient air quality standards from the United States environmental protection agency (EPA) (35 *μ*g/m^3^) and the World Health Organization (25 *μ*g/m^3^). In two venues, the airborne PM_2.5_ was around 1500 *μ*g/m^3^, roughly half the occupational exposure guideline for respirable particulate matter,^20^ suggesting that worker protection measures need to be considered, including personal monitoring, hazard communication, and control implementation if personal samples exceed occupational exposure guidelines. PM_2.5_ concentrations in Baltimore water pipe venues were approximately twice as high as those measured in 17 waterpipe cafes from Virginia during March and May 2011 (mean PM_2.5_ concentration of 374 *μ*g/m^3^).^15^ PM_2.5_ concentration in Baltimore waterpipe venues were roughly half of those reported in a recently published study of waterpipe cafes in Canada (1419 mg/m^3^).^16^ PM_2.5_ concentrations in the Baltimore waterpipe venues exceeded PM_2.5_ concentrations measured in hospitality venues that allow cigarette smoking.^21, 22, 23^

Waterpipe emission testing has indicated that side-stream smoke from a single waterpipe session produces up to 30 times the CO as a smoked cigarette.^8^ In our study, average CO concentrations in four of the six venues visits exceed the EPA 8-h CO air quality standard of 9 p.p.m.. The overall average CO concentration across all visits was twice the EPA 8-h standard. In one of the sampling visits, Venue #5, the average CO concentration exceed the EPA 1-h standard of 35 p.p.m. for the entire sampling time. Average CO concentration measured at Venue #5, 53 p.p.m., also exceeded the recommended occupational threshold limit value for CO of 25 p.p.m. during an 8-h period.^20^ The critical effects for this standard include anoxia and effects on the cardiovascular, central nervous, and reproductive systems.^20^ The high CO exposure concentrations found in water pipe venues represent a significant occupational exposure hazard to workers in the waterpipe bars. Personal sampling is needed to more accurately assess exposures. If these exposures are found to exceed exposure guidelines, steps to reduce exposure should be implemented.

The airborne nicotine results we obtained were generally lower than assessments in bars and restaurants that allow cigarette smoking. Airborne nicotine concentrations in restaurants and bars that allow smoking range from 1 *μ*g/m^3^ to ∼20 *μ*g/m^3^.^19, 24^ For example, the median airborne nicotine concentration from a comprehensive international study of 467 bars and nightclubs collected over a 7-day period was 3.5 *μ*g/m^3^ with an interquartile range of 1.5 *μ*g/m^3^ to 8.5 *μ*g/m^3^.^19^ The airborne nicotine results in our study in waterpipe venues were below 2 *μ*g/m^3^. In a study conducted in 12 waterpipe cafes from Toronto, mean air nicotine concentrations collected during a 2-h period was 3.3 (SD 2.7) *μ*g/m^3^.^16^ Additional studies with a wider sampling of waterpipe venues and longer sampling durations are needed to confirm whether air nicotine concentrations in waterpipe cafes are similar or lower compared with venues with cigarette smoking.

Waterpipe smoking was likely the major source of PM_2.5_, CO, and nicotine measured because the majority of the venues had no alternate sources of combustion byproducts such as cooking, open fires, or cigarette smoking. The charcoal used to heat the waterpipe tobacco is clearly an important source of PM_2.5_ as well as CO. PM_2.5_ and CO concentrations were highly correlated across waterpipe venues. Additional research is needed to confirm this correlation in a larger sample.

The Sidepak measures real-time PM based on light-scattering properties and is manufacturer-calibrated using Arizona road dust. Light-scattering properties of particulate matter will vary depending on the size distribution, reflectance, and the aerosol composition. Therefore, it is important to apply a correction factor generated from gravimetric PM measurements of the aerosol being sampled to the Sidepak measurements. Our study is the first to apply a field-determined calibration factor to the Sidepak measurements to evaluate waterpipe secondhand smoking constituents in a real world setting. The calibration factor determined in this study, 0.6, is in general agreement with a previously reported value of 0.4.^15^

Study limitations include the short sampling time (2–5 h) and small sample size. In addition, all concentrations reported in this paper are based on area sampling and may not necessarily represent actual personal exposures. In addition, we did not sample for 8 h, nor did we use an occupational sampling convention for PM (i.e., respirable dust), so it is problematic to compare our results to occupational standards or guidelines. Future studies should include full-shift personal samples using occupationally relevant size-selective samplers for PM to assess compliance with occupational standards and guidelines. In addition, we did not directly assess ventilation or air exchange rates within the venues making it difficult to explain the indoor air concentration variability within and between venues. These data support the need for further studies that include a larger number of venues, personal air sampling, direct ventilation assessment, and biological monitoring of patrons and employees in waterpipe venues.

This study adds to the small but growing literature indicating that both non-smoking employees and patrons of waterpipe venues are at increased risk from complex exposures to secondhand waterpipe smoke. The indoor concentrations of both PM_2.5_ and CO exceeded health-based recommendations and were markedly greater than expected compared with venues allowing cigarette smoking. These findings contribute to the understanding of waterpipe smoke as a hazardous indoor pollutant, at least as toxic as cigarette smoke. Workers and customers in waterpipe venues may be exposed to extremely high concentrations of regulated hazardous pollutants.

## Figures and Tables

**Table 1:** Venue characteristics and waterpipe smoking observations.

*Venue ID* a	*Area (volume)*	*Number of people*		*Number of lit waterpipes*		*Number of people actively smoking waterpipes*		*Average number of people sharing waterpipes*		*Active waterpipe density* b
	*m^2^ (m^3^)*	n^c^	*Mean (SD)*	N^c^	*Mean (SD)*	N^c^	*Mean (SD)*	N^c^	*Mean (SD)*	*Mean (SD)*
1-A	32 (112)	15	18 (13)	15	13 (7)	14	14 (10)	1	6 (0)	12 (9)
1-B		17	22 (12)	17	12 (8)	17	11 (7)	17	6 (2)	10 (6)
2	45 (225)	12	10 (2)	12	14 (5)	9	8 (5)	4	4 (1)	4 (2)
3-A	44 (192)	11	16 (13)	12	14 (8)	12	11 (7)	10	3 (1)	6 (4)
3-B		10	16 (3)	10	10 (7)	10	15 (6)	9	5 (1)	8 (3)
4	119 (476)	12	41 (2)	12	13 (4)	—	—	—	—	—
5	140 (490)	13	21 (13)	12	12 (5)	12	9 (5)	13	4 (1)	2 (1)
6	119 (476)	12	26 (8)	12	14 (9)	12	11 (6)	12	8 (1)	2 (1)
7	119 (298)	9	33 (18)	9	12 (4)	9	9 (3)	9	5 (1)	3 (1)

—, No observations recorded.

a Multiple visits to venues denoted with the suffixes –A and –B.

b Active waterpipe density: average number of people actively smoking waterpipes per 100 m^3^.

c *n*=number of observations at each venue (collected at approximately 15-min intervals).

**Table 2:** Secondhand smoke constituents–Carbon Monoxide, PM_2.5_, and airborne nicotine.

*Venue ID* a	*1-A*	*1-B*	*2*	*3-A*	*3-B*	*4*	*5*	*6*	*7*	*Overall*
*Carbon monoxide, p.p.m.* b										
Mean (SD)	15 (7)	19 (17)	2 (2)	7 (4)	—	—	53 (30)	2 (2)	—	18 (23)
Median (Interquartile range)	12 (9–24)	22 (1–34)	2 (0.25–3)	8 (2–10)	—	—	51 (33–72)	2 (1–4)	—	9 (2–25)
Maximum	32	60	7	14	—	—	115	7	—	115
										
*Real-time PM*_*2.5*_, μ*g/m*^*3*^										
Mean (SD)	235 (309)	732 (767)	187 (282)	1594 (788)	788 (540)	320 (186)	1561 (938)	676 (343)	72 (77)	712 (785)
Median (Interquartile range)	129 (71–243)	554 (78–991)	88 (30–175)	1788 (1158–2133)	682 (375–1020)	279 (215–353)	1322 (961–2463)	635 (390–942)	43 (6–128)	374 (127–1032)
Maximum	1737	4907	1610	3134	3799	1616	3628	1627	291	4907
										
*Airborne nicotine,* μ*g/m*^*3*^										
Mean	1.22	1.66	0.77	1.90	1.86	0.97	1.13	1.84	1.47	1.42^c^

a Multiple visits to venues are noted with the suffixes –A and –B.

b Values below the level of detection replaced with ½ level of detection, or 0.25 p.p.m.

c Arithmetic mean of all samples.

**Table 3:** Correlation between PM_2.5_ and carbon monoxide.

*Venue ID* a	n	*Correlation coefficients PM*_*2.5*_ *and CO*
1	462	0.77
2	183	0.87
3	173	0.72
4	—	—
5	218	0.97
6	173	0.77
7	—	—

*n*=number of observations.

— Both PM_2.5_ and CO values were missing.

Spearman correlation coefficient; all *P*-values were <0.001.

a For venues 1 and 3, data from multiple visits included.
