# Supplementary material for: Waterpipe cafes in Baltimore, Maryland: Carbon monoxide, particulate matter, and nicotine exposure
Source: J Expo Sci Environ Epidemiol. 2014 Apr 16;25(4):405–10. doi: 10.1038/jes.2014.19 (PMC4333110; doi:10.1038/jes.2014.19)
Supplement: Supplementary Table S1 [file jes201419x1.doc]

**Supplement Table 1. Comparison of Carbon Monoxide and Particulate Matter in Outdoor vs. Indoor Air**

|  | PM_2.5_ (µg/m^3^) | | CO (ppm) | |
| --- | --- | --- | --- | --- |
| Venue ID (a) | Outdoor (a) | Indoor (b) | Outdoor (a) | Indoor (b) |
| 1-A | 153 (351) | 235 (309) | 10 (11) | 15 (7) |
| 1-B | 247 (302) | 732 (767) | 12 (15) | 19 (17) |
| 2 | 144 (198) | 187 (282) | 0.40 (0.36) | 2 (2) |
| 3-A | 115 (304) | 1594 (788) | 0.96 (2.3) | 7 (4) |
| 3-B | 415 (647) | 788 (540) | -- | -- |
| 4 | 85 (89) | 320 (186) | -- | -- |
| 5 | 41 (13) | 156 (938) | 6 (7) | 53 (30) |
| 6 | 28 (7) | 676 (343) | 0.69 (0.77) | 2 (2) |
| 7 | 27 (35) | 72 (77) | -- | -- |
| Values are mean (SD). | | | | |
| (a) Multiple visits to venues denoted with the suffixes –A and –B. | | | | |
| (b) Outdoor samples collected approximately 10-15 minutes before entry and after exit from venue, directly in front of the entrance. | | | | |
